# Experiencing accessibility of historical heritage places with individuals living with visible and invisible disabilities

**DOI:** 10.3389/fresc.2024.1379139

**Published:** 2024-04-03

**Authors:** Alicia Ruiz-Rodrigo, Ernesto Morales, Maryem Lakoud, Jonathan Riendeau, Miranda Lemay, Ariane Savaria, Samuel Mathieu, Isabelle Feillou, François Routhier

**Affiliations:** ^1^ School of Rehabilitation Sciences, Université Laval, Québec City, QC, Canada; ^2^ Centre interdisciplinaire de recherche en réadaptation et intégration sociale, Centre intégré universitaire de santé et de services sociaux de la Capitale-Nationale, Québec City, QC, Canada; ^3^ Industrial Relations Department, Université Laval, Québec City, QC, Canada

**Keywords:** accessibility, environment, go along interview, walking interview, people with disabilities, inclusion, participation, built heritage

## Abstract

**Introduction:**

Around 16% of world's population lives with visible and invisible disabilities. People with disabilities' participation may be limited because of the environmental obstacles. Moreover, historic heritage places were built before the development of accessibility standards and the rights of people living with disabilities and the majority were not designed to be accessible. Access to historic heritage places is important for carrying out the activities in place but also to create and reinforce identity. The aim of this study was to explore the experiences of people with visible and invisible disabilities when visiting heritage sites considering accessibility issues.

**Methods:**

This study is a qualitative interpretive description. Participants were adults with visible (e.g., motor disability) or invisible (e.g., autism) disabilities. For data collection, go along interviews (also referred to in the literature as “walking interview” in two different locations in the Historic District of Old Quebec in Quebec City were conducted. Thematic analysis was done.

**Results:**

Twenty-one participants completed two go along interviews: one in the *Séminaire de Québec* (Seminary of Quebec City) and the other in Petit-Champlain and Place Royale areas of Quebec City. Three themes emerged: (1) Obstacles and impact on participation; (2) Disabling accessibility; and (3) Heritage meaning.

**Discussion:**

The barriers identified by participants are diverse and differ according to the person and the type of disability. However, social and leisure activities were particularly limited, despite the strategies developed by some participants. Participants in the study demonstrated an interest in accessing to heritage places, therefore it seems essential to consider the needs of people with disabilities when developing accessibility solutions, and to seek a balance between preserving heritage and promoting inclusive and equitable access for all.

## 1 Introduction

People with disabilities currently represent around 16% of the world population (1). Both people with visible (e.g., wheelchair user) and invisible (e.g., autistic person) disabilities may experience challenges related to environmental barriers that may hinder their social participation. The concept of social participation refers to “the total accomplishment of life habits, resulting from the interaction between personal factors (impairments, disabilities and other personal characteristics) and environmental factors (facilitators and obstacles)” (2). According to the Invisible Disabilities Association, an invisible disability is a “physical, mental or neurological condition that is not visible from the outside, yet can limit or challenge a person's movements, senses, or activities” (3). The elements of the environment involved in social participation are very diverse, and participation can therefore be affected at different levels and in different spheres of the person's life.

Many people with disabilities experience accessibility problems at public spaces. This means that the person may have difficulties entering the building or may not be able to enter at all. In other situations, even if the person is able to access the building, it may not be possible for the person to complete the intended activities in that location (4). Among public spaces, historic places, which were built before the development of accessibility standards and rights for people living with disabilities are often some of the most inaccessible. As they were not designed to be accessible (5), some people with disabilities find them difficult to access and navigate (4).

According to *Canada's Historic Places,* a historic heritage place is “a structure, building, group of buildings, district, landscape, archaeological site or other place in Canada that has been formally recognized for its aesthetic, historic, scientific, cultural, social or spiritual importance or significance for past, present or future generations (heritage value)” (6). Enhancement and transmission of cultural heritage is important because it is a reflection of a society's identity (7). But also, historic heritage places have different functions associated to several categories such as residence, education, health and research, religion, community, government activities or transportation, among others (8). Since historic heritage places have various functions, many activities such as tourism and cultural leisure, education, work, participation in the political life, may be restricted for people with disabilities (9). Moreover, heritage places are usually protected, and they cannot be modified, so adaptations to make them accessible constitute a significant challenge. Therefore, historic heritage places can be particularly problematic environments for people with disabilities as they are sometimes partially accessible and usually inaccessible (10). Some examples of the most frequently reported barriers to access to these kind of places are steps at the entrance or inside the buildings, the lack of handrails, the uneven floors, the sidewalks or their absence, sound reverberation, the lack of lighting, complexity of presented information (ex.: long texts or complex language), and insufficient space in bathrooms (9, 11–15).

Although there is some literature about access to culture that takes into consideration the point of view of people with disabilities, such museum accessibility (16, 17), there are not so many studies that have been carried out in the specific field of historical heritage considering the first-person experience of people with disabilities (10, 12, 15, 18). Studies that consider the perspectives of people with disabilities in a patrimonial context usually do so through interviews or questionnaires (9, 19) or usually only involve people with physical disabilities (20, 21). Thus, the propose of this study was to explore the experiences of people with visible and invisible disabilities when visiting historic heritage places considering accessibility issues.

## 2 Methods

This study used a qualitative interpretative description approach (22) to understand people living with disabilities' experiences. In order to explore and describe experiences in a real context, go along interviews method in heritages places was used. This technique is usually referred to in the literature as “walking interview” (23, 24). However, in view of the differences in the mobility abilities of the study participants, the term “go along interview”, will be used throughout this article. This term, already used in some studies (25), is less ableist and focuses on the characteristics of the interview rather than on the individual characteristics and functioning. Research team members come from a variety of backgrounds and disciplines, such as occupational therapy, architecture, design, sociology, and engineering, including accessibility and heritage experts. A multidisciplinary team enables the combination of knowledge from different fields, which is essential for the study of accessibility of historic heritage places and the development of research studies adapted to the needs of the context (26, 27).

### 2.1 Participants and recruitment

Participants were adults with disabilities responding to the following selection criteria: (1) to live with a visible (motor, visual, normal aging process related) or invisible disability (autism, intellectual disability, hearing disability, chronic pain or fatigue); (2) to be 18 or older; (3) to be able to communicate with the research team with or without aids or support; (4) to be able to get to and navigate in the Old Québec (Vieux-Québec) Historic District, in City of Quebec (Canada), with or without mobility aids. Although people with invisible disabilities may sometimes have certain traits that are recognizable to others, they often go unnoticed in casual interactions. However, their quality of life and functionality can be as much affected as for people with more obvious disabilities (28). People with invisible disabilities “needs cannot be accommodated simply by making “obvious” physical alterations in the structures of ingress and egress, but only by making more sweeping changes in the environment” (28). For this reason, it is essential to include this population in the study.Snowball and convenience sampling was carried out (29). A recruitment poster including a description of the study and the interview process, as well as eligibility criteria, was distributed to various Facebook groups of people with disabilities. Several organizations related to the targeted disabilities (e.g., *Regroupement d’Organismes de Personnes Handicapées de la region 03* - ROP 03; Kéroul, Bureau des étudiantes en situation de handicap de l'Université Laval) participated to the recruitment process. Some participants from the researchers' participant databases were also contacted to complete the recruitment to ensure the participation of people with all sorts of disabilities. Participants who had expressed an interest in taking part of the study were contacted by telephone or e-mail to determine if they were eligible and to explain the project and their participation. No diagnostic documents were required to participate, the person self-identified as living with a disability.

### 2.2 Data collection

For the data collection, the team members conducted go along interviews (23, 24) in the Historic District of Old Quebec in Quebec City, included in the UNESCO World Heritage List since 1985 (30). This technique consists of on-site interviews, in which participants can explain their experiences in relation to a specific environment while visiting the place. As person-environment interaction may be “difficult to express in simple terms” (31), it facilitates the expression of the perceptions concerning the environment thanks to the real-time and direct interaction between the person and the environment (24, 32). This method also allows researchers to perceive the attitude and behavior of the person as well as the changes of the environment.

**Figure 1:**
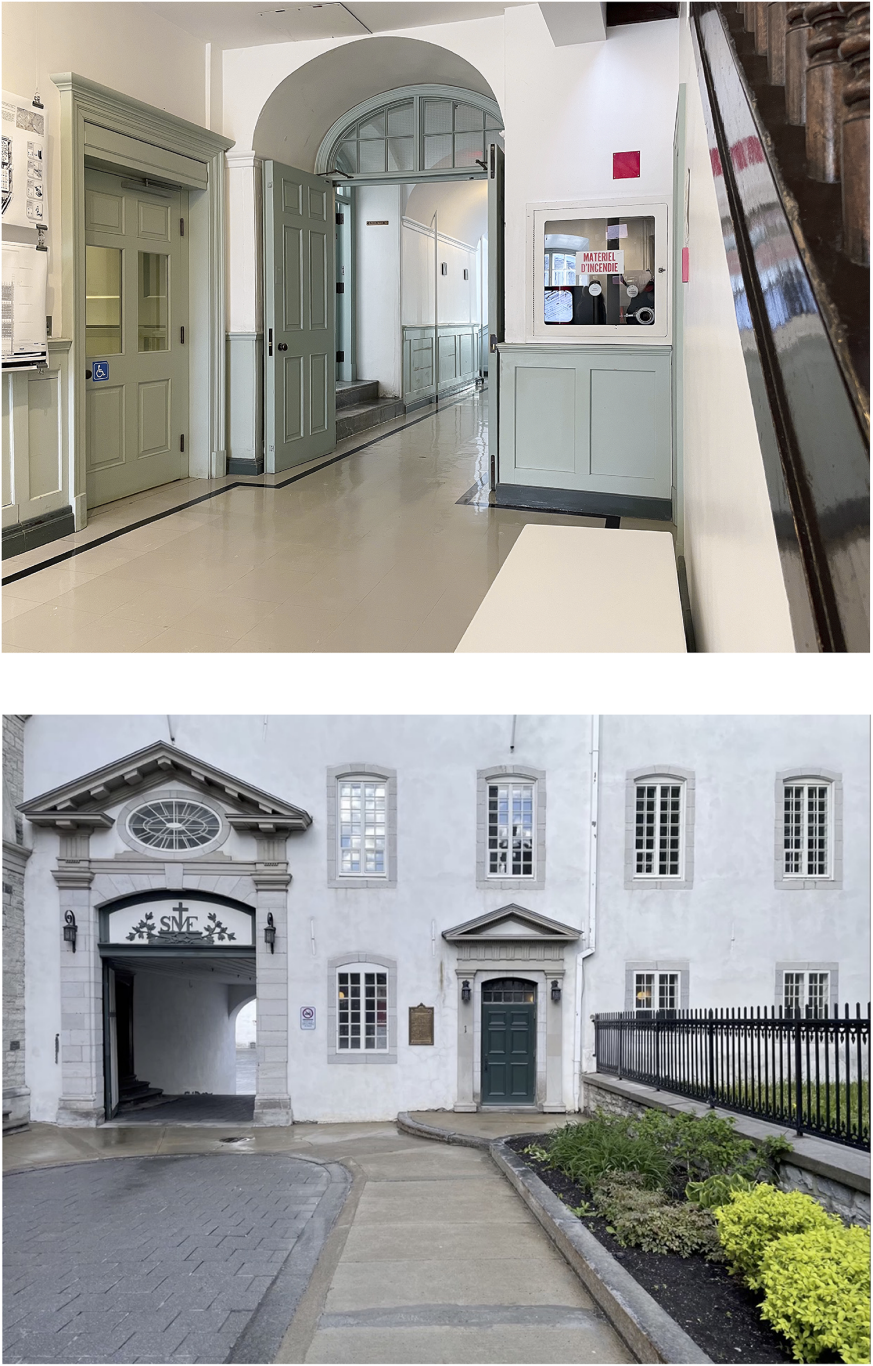
Séminaire de Québec.

**Figure 2:**
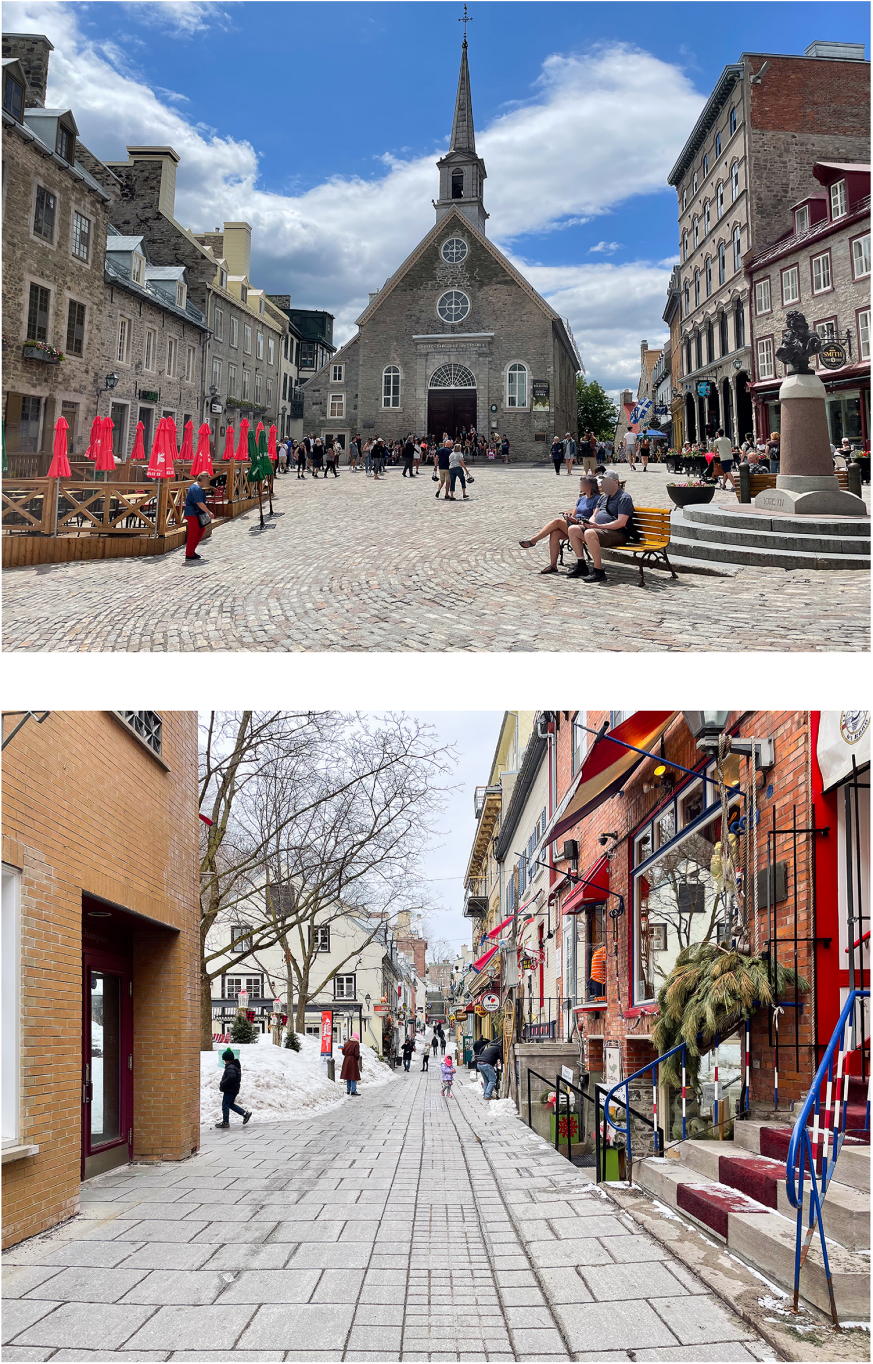
Petit-Champlain and Place Royale sectors.

Two historic heritage places were chosen for the go along interviews: (1) School of Architecture of Université Laval located at the *Séminaire de Québec* (Seminary of Quebec) (Figure 1) and (2) Petit-Champlain and Place Royale sectors in the Old-Québec area (Figure 2). Both places were chosen because of their heritage-related importance. *Séminaire de Québec* is a building and the other one Petit-Champlain and Place Royale sectors are exterior sites, so both, indoor and outdoor historic heritage places could be explored. Also, both places allow participants to visit them with members of our research team even if there are some environmental barriers. *Séminaire de Québec* has some adaptations which allow people with mobility impairments' circulation in most of the floors, such an elevator and two lifts, and some adapted toilets, which does not mean they were completely accessible. The itinerary in Petit-Champlain and Place Royale sectors did not include adaptations, but circulation on certain streets was possible for participants. The itineraries were predefined in order to ensure the safety and comfort of the participants as well as the study of environments presenting heritage characteristics. Two or three members of the research team, with no relationship established with participants prior to study, were present during the go along interviews. One of them led the interview and guided the participant orally (a sign language interpreter was provided for the interviews with a deaf person), and the others checked technical elements (e.g., microphones, recorder, and camera) and managed contextual issues (e.g., taking the library's key when it was closed). All participants had to complete two go along interviews, one on each site, and this was done between April and August 2022 during daytime or evening to cover different environmental situations along with different weather conditions. Some Covid-19 travel measures, such as proof of vaccination, were still in place during the data collection period. Participants had to describe their experiences in both places and asked some open questions based in a semi-structured interview guide. Interviews were both filmed and recorded for subsequent transcription and analysis.

### 2.3 Data analysis

Every go along interview was integrally transcribed by a team member, and the transcripts were revised by the first author to ensure accurate transcription (ARR). Videos were used to contextualize audio data when necessary (24). Two cycles of analysis were carried out. First, coding (33, 34) was conducted to structure the data and guide subsequent analyses. A codebook which was prior developed was used and it was based on two sources: (a) the Human Development Model—Disability Creation Process (HDM-DCP) (35, 36), and (b) on some elements of the Rick Hansen Foundation Accessibility Certification (RHFAC) survey (37). The codes referred to the person, the social and physical environment, and the activities and roles. HDM-DCP Model provides a perspective on the interaction between the person and the environment. The Rick Hansen Foundation Accessibility Certification (RHFAC) survey provides a structure for addressing potential physical environmental obstacles. Five members of the research team participated in the coding process (ARR, MLa, JR, MLe, AS). Each research team member involved in the coding process individually coded the same two complete interview transcripts (one outdoor interview and one indoor interview). Then the team members carried out two team meetings in order to homogenize and calibrate the understanding of each code as well as the criteria in the choice of codes. After this coding validation process, every interview transcript was coded by one person of the team and revised by another team member. All coding was performed on NVivo version 13 (38). Secondly, once the first coding was complete, theming the data analysis (34) was carried out to identify participants' experiences. Then, similarities and differences among the emerging elements have been explored and discussed within the team in order to identify and organizing themes and sub-themes. To minimize bias possibilities, the research team carried out discussion sessions throughout the entire analysis process.

### 2.4 Ethics

The study was approved by the sectorial ethics committee on research in rehabilitation and social integration of the *Centre intégré universitaire de santé et de services sociaux de la Capitale-Nationale* (#2022-2422) and every participant signed a consent form.

## 3 Findings

Twenty-one individuals participated to this study and each one completed two go along interviews, one at the *Séminaire de Québec* and one at Petit-Champlain and Place Royale sectors, for a total of 42 go along interviews. Most participants were not familiar with the *Séminaire de Québec*, although some had already visited the Petit-Champlain and Place Royale areas. Regarding participants' characteristics, 57.1% (*n* = 12) of the participants were women, 38.1% (*n* = 8) were man and 9.5% (*n* = 2) was nonbinary people and the ages of the participants ranged from 22 to 79 years old (median = 40). Participants had different disabilities and different functional profiles, 52.4% (*n* = 11) live with a visible disability and 47.6% (*n* = 10) with an invisible disability. Most participants had co-morbidities, meaning that they also had other types of disability, often milder than the main one. All the participants who used assistance devices were familiar with them, except for one who had recently obtained her mobility device (walker). Concerning perceived limitations, most participants felt limited by environmental barriers daily, but the perception of the frequency of limitations was heterogeneous within participants (see Table 1 for detailed participants' characteristics and Table 2 for assistive devices and support information). The go along interviews lasted between 27 and 125 min. Every participant finished the interview at the *Séminaire de Québec* and only one of the participants could not finish the interview at Petit-Champlain and Place Royale sectors due to the pain while navigating the uneven pavement (the interview lasted 8 min).

**Table 1:** Participants’ characteristics (*n* = 21).

Age	*n*	%
22–35	7	33.3
36–50	3	14.3
51–64	4	19.0
65–79	5	23.8
Did not provide age	2	9.5
Gender*		
Woman	12	54,5
Man	8	36.4
Non-binary	2	9.1
Main disability		
Mobility	4	19.0
Vision	4	19.0
Hearing	2	9.5
Autism	4	19.0
Intellectual	1	4.8
Pain	2	9.5
Aging-related	4	19.0
Comorbidities		
Yes	14	66.7
No	7	33.3
Limitation frequency		
Everyday	13	61.9
Several times a week	2	9.5
Several times a month	1	4.8
Several times a year	2	9.5
Never	0	0
Did not provide limitation frequency	3	14.3

* *n* = 22 for this characteristic: a person has indicated that she was both a woman and non-binary.

**Table 2:** Assistive devices and support.

Main disability	Assistive device or support
Mobility (*n* = 4)	Manual wheelchair (*n* = 2)
	Motor wheelchair (*n* = 1)
	Walker (*n* = 1)
Vision (*n* = 4)	Dog (*n* = 1)
	White cane (*n* = 2)
	None (*n* = 1)
Hearing (*n* = 2)	Interpreter (*n* = 1)
	Cochlear implant (*n* = 1)
Autism (*n* = 4)	Dog (*n* = 1)
	None (*n* = 3)
Intellectual (*n* = 1)	None (*n* = 1)
Pain (*n* = 2)	Motor wheelchair (*n* = 1)
	None (*n* = 1)
Aging-related (*n* = 4)	None (*n* = 4)

The three themes that emerged from the data analysis were: (1) Obstacles and impact on participation: which addresses the obstacles encountered by participants during the go along interviews, their impact on participation, the strategies developed by participants, as well as the roles of other individuals present on the site; (2) Disabling accessibility: which includes some of the partial accessibility solutions already present on sites, and the feelings experienced by participants towards these solutions, and (3) Heritage meaning: addressing the meaning and importance of historic heritage for people with disabilities. Citations are meant to identify the number of participant, gender initial, and condition (e.g., autism, mobility, etc.).

### 3.1 Obstacles and impact on participation

Most participants had already visited Old Quebec simply to walk around and enjoy the atmosphere, for cultural activities or events (e.g., workshops, shows, carnival, theater) or to go to local restaurants and bars. However, they explained how their participation in these activities could be hindered by obstacles such as uneven ground, sidewalk obstructions (e.g., shop displays, other posters, garbage cans) or the lack of rest areas and street furniture. A participant mentioned: “That [pavement] is something I hate in my life. I don't like it (…) But you can't change it. There are a lot of cities like that in the world” (P07M-Mobility).

Other factors that could limit participants' activities would be echoes, too many sensory stimuli or crowded conditions. Echo, a characteristic feature of heritage buildings, was mentioned by several participants with different disabilities as an obstacle. Notably autistic people and those with hearing impairments, but also participants with visual disabilities or chronic pain considered it to be disruptive or distracting, and a significant barrier to communication. A participant said:

When there's an echo, it's hard for me to understand. The voices seem to blend together, and it's hard for me to situate myself. And I can only hear on one side, so I can't localize the sounds. I have the impression that the sounds are coming from everywhere. (P15W-Hearing)

Concerning sensory stimuli, in particular, participants with autism mentioned that there were too many sensory stimuli, especially on the outdoor course and in terms of visual and acoustic stimuli. The variety of colors, shapes, and textures, as well as noise and music, were elements that could make a visit to Vieux-Québec uncomfortable for some of the participants. One participant explained: “So, it is a lot… (…) It's like: o.k. There's a lot of smells, there's a lot of people, there's a lot of noise” (P18W-Autism). In terms of crowds, since Petit-Champlain and Place Royale are tourist areas, large groups of people on site were disruptive for many participants. For example, a participant reported that a group around a street musician could be disturbing for deaf people because they might have difficulty understanding the situation as they don't perceive the sound information. But also, for autistic people who could feel overload. Concerning shows and cultural events, one participant mentioned that these were not always adapted to the deaf community and suggested varying the types of events and including deaf-friendly activities such as mimes:

The square is open, so I can see everything. Sometimes, when there's a show, people gather around. But we can't hear. There might be people laughing. There's a joke … Sometimes there are little gatherings like that in the summer, eh? Little performances. We don't hear them, so we look to see if there's ever any action, like, more, mimes, or uh … That would be interesting, we'd be more likely to stay, at least, to enjoy the activity. (P16W-Hearing)

Access to toilets was often raised as a potentially problematic feature. Several participants, with different disabilities, explained that this would be one of the first things they would like to spot when visiting a new location. However, some participants said they avoided going to public toilets for various reasons (e.g., lack of cleanliness, toilets only partially accessible). One participant even mentioned that some people with disabilities will resist drinking before going to a place where they are not guaranteed easy access to toilets: “When I get home, I drink two or three glasses of water, I'm thirsty because we try to avoid drinking. (…) Because it's a bit annoying to always be looking for an accessible bathroom” (P06W-Mobility). Additionally, access to shops and restaurants was often limited, for instance due to steps or an inaccessible entrance, lack of toilet facilities on the floor and difficulties in communicating with staff.

To compensate for these obstacles, the majority of participants explained the strategies they had consciously or unconsciously developed alternatively.

#### 3.1.1 Participants' strategies

Many different strategies, depending on the person and the type of disability, were mentioned by participants. Many participants mentioned having to plan their activities in advance to ensure that environmental conditions would allow them to carry out the activities once on site. To do this, some participants explained that they consulted the city or district website, as well as pages or groups of people with disabilities on digital social networks. A participant mentioned:

The planning process … I'd go on the Internet, on the Quebec City website, uh, and then I'd go to the … the neighborhoods or the districts of the city. I'd go to the Petit-Champlain district, then I'd look at what's there, stores, and restaurants. There are stairs to the top. There's the château at the top. So, I'd look at the little attractions around it. (P12W-Intellectual)

Regarding the excess of stimuli, autistic participants also mentioned several strategies such as using earplugs, focusing only on one element of the environment (“tunnel vision”), favoring already familiar places, avoiding busier areas, and settling in quiet places (e.g., rest area) to reduce the presence of stimuli. A participant noted:

You know, it doesn't take much, it doesn't take a room, an isolation room, necessarily … But a corner, you know, somewhere. (…) I would sit down, put on my earplugs so I can't hear anything, and wait for it to pass, you know. Because … there's a lot of people there, because there's noise, because there's … You know, there's a lot of things there. (P18W-Autism)

Several communication strategies were mentioned. On one side, some participants consulted maps on their cell phones, or read text (in the case of people with visual disabilities) by taking a photo and using the “VoiceOver” option, which provides descriptions and screen-reading. On the other side, using paper and pencil to communicate when needed was the strategy used by the deaf person. For carrying out activities, she mentioned that she had to engage in group activities and hire a sign language interpreter out of their own money, since leisure activities are not considered essential (unlike medical appointments, for example, where the interpreting service would be provided): “We'd already booked a glassblowing workshop, glass that we heat. Then we had booked an interpreter, to go to the workshop that we had booked to come here, in town” (P16W-Hearing).

In addition, when signs or directions were unclear or it was difficult to find one's way around, participants had two choices: make additional trips or ask for help. Some participants explained that they would often avoid interactions with other people, particularly requests for help, and would therefore prefer to find solutions on their own.

Well, one of the reasons I like having a map like this is that I don't have to ask people around me. (…) It's harder for me to go looking for information, to ask “hey, where's that store?" I like having the map so I can find my way around without having to communicate with someone I don't know. (P15W-Hearing)

However, as navigating was often difficult for participants in this type of environment, some people mentioned that they will, sometimes in spite of themselves, ask for help from people present on the premises to avoid moving around more than necessary.

#### 3.1.2 People, a facilitator or a barrier?

Heritage places are often tourist or busy places, and the presence of other people would seem to have an impact on participation for many participants. While some noted that contact with other people was positive for them, many of our participants mentioned that crowds could become an obstacle for various reasons. Participants with different types of visible and invisible disabilities (wheelchair users and other mobility aids, people with autism, people with intellectual disabilities, people with age-related disabilities, visual disabilities, and hearing disabilities), mentioned problems, obstacles, or discomforts in this regard. For example, some people with different disabilities (e.g., hearing, mobility, autism), mentioned that crowds could easily block circulation or make communication difficult: “That's for sure, because it's harder for me to understand [other people] when there are a lot of people around me. But I like it too, in a way, because it makes things animated, you know, it makes … It adds life” (P15W-Hearing). Participants who had an assistance dog also mentioned that other people and other dogs might tend to interact with theirs. Others mentioned that they felt stressed in crowds or would have feared getting lost if they had found themselves alone in a crowd. As one participant pointed out: “The biggest obstacle? The biggest obstacle … Uh … Not getting lost in the crowd when there's a lot of people around” (P12W-Intellectual). In addition, some participants explained that they would appreciate the presence of quiet rest areas. However, several participants mentioned that the people around them did not represent an obstacle for them, at least with the level of crowds existing during the go along interviews.

In several situations during the go along interviews, and according to anecdotes shared by some participants, they perceived that individuals without disabilities would be more aware of accessibility issues than in the past. One participant shared a situation she and a group of deaf people had experienced in a restaurant in the Petit-Champlain district:

We were able to order, um … Of course, sometimes you don't understand, you know, you try to read lips. Then finally, with the printed text, it worked … We put our thumbs up. We said: “Yes, that's good”. The waitress at the table was open-minded. (P16W-Hearing)

Nevertheless, some participants mentioned that they felt more at ease when they were accompanied around historic heritage sites, which they felt was more relevant on a first visit, especially if they have a visual impairment. Others said that being accompanied made their orientation easier, or reduced their stress levels: “Right now, yeah, I feel … good …. I feel well accompanied. Being on my own, I don't know (…) I'd be able to do it on my own, but I'd be more stressed” (P21M-Autism). Despite this, most participants expressed their interest in being able to enjoy historic heritage places completely by themselves, which could be encouraged with the implementation of accessibility solutions.

### 3.2 Disabling accessibility

In historic heritage places, some of the major obstacles identified by participants were characteristic features of the historic environment, such as irregular pavement, heavy doors, echoes in indoor settings, complexity of the building's structure or the presence of steps at building entrances. Accessibility solutions are sometimes available to counter these obstacles, but they do not always enable complete autonomy. An example of this type of solution would be those requiring interaction with another person or the presence of an assistant or another person (e.g., platform lifts requiring a member of staff to activate it with a key, or removable ramps). Confronted with this kind of solution, one of the participants commented: “It's just really *ableist*, as they say. To say, like, we can't go out unless we’re accompanied with another person. If we don't have anyone, we stay home (…) It's a bit insulting” (P13W-Pain). In a normal situation, such a situation could have led to feelings of insecurity or to concrete situations of discomfort or risk for people with disabilities.

#### 3.2.1 Comfort and safety

People with disabilities seem to sometimes experience situations of physical or social discomfort, as well as feelings of insecurity or risk situations. For example, with regard to feelings of insecurity, two participating women mentioned that they wouldn't necessarily feel safe if they did the route alone and at night (P06W-Mobility, P12W-Intelectual). This feeling of insecurity was more related to her gender than to her disability. In terms of discomfort, access through secondary doors—often the only adapted ones—would be socially and dignity-disturbing for people with mobility disabilities. Another uncomfortable situation would have been to wait for the physical environment to be adapted for access, for example due to the installation of an accessibility solution or simply the opening of a double door to make the space wider. This can also make the person feel observed by others in the area. For example. during one go along interview, the participant was trying to access a business whose manager explained that the second door could be opened. The participant mentioned that she felt like she was in the spotlight at that time (P06W-Mobility).

In terms of safety and physical comfort, elements such as the pavement, the slope of the ground or poorly-maintained sidewalks can make navigating very uncomfortable or even unsafe for the person. These circumstances led one participant, who uses a wheelchair and lives with chronic pain, to stop her outdoor go along interview. Other participants with the same type of disability (chronic pain) also expressed discomfort when moving around on this kind of surfaces: “Especially as the floor isn't, uh, it's not super smooth, there are cracks. So sometimes, you know, if you don't have very strong ankles or good balance, it would be easy to get stuck” (P09W-Pain).

Despite the difficulties experienced during the go along interviews, and the lack of accessibility of the heritage sectors visited, participants expressed their interest in heritage and highlighted the significance these places had for them.

### 3.3 Heritage meaning

Most participants, regardless of their type of disability, associated heritage with history, beginnings or origins, “what the ancestors left behind” (P02M-Visual), but also with culture and a socio-political context. Some participants saw heritage as a source of baggage and reference points for society: “So it's a document that's very faithful (…) And it reflects where we come from. If you know where you come from, there's a good chance you know where you're going” (P11M-Aging-related).

In addition to emphasizing the beauty of the sites, participants perceived historic heritage places as special places with character and a soul, which are important to experience. Often, participants associated the places with the most historic heritage features with a pleasant, warm atmosphere. For example, one autistic participant described the library he visited in the *Séminaire de Québec* as a warm and comforting place:

Do you know that my comrades are likely to end up here? You know, I feel like I'm describing a different kind of animal, but people who are hyper-sensitive or have disorders like that, not just ASD, we like quiet places. And here, it's like a comforting place, where I would tend to come back often (…) I get dizzy imagining all the treasures that are hidden behind the doors, just waiting to be discovered. (P14M-Autism)

Participants described their understanding of heritage and how important it was to them. Despite accessibility issues, all participants showed an interest in heritage and especially in the possibility of accessing it, even if some did not identify themselves as regular museum visitors or as having a great interest in history: “Heritage? Yes, well, I'm not a big fan of museums, but still, I think it's important. Yes, it's important to go and see things from the past, it helps you see how things used to be” (P2M-Visual). Another participant said:

I recognize how important it is, but for now it's a statement, not a life's habit. I like it when I'm invited to a museum. On my own initiative, I don't go (…) I say to myself: “Starting next month, I'm going to start going to the theater, and museums” every time I go, I'm transformed (…) like, “Wow, this has deepened my understanding of the land of my ancestors”. (P14M-Autism)

#### 3.3.1 Balance between accessibility and heritage

The majority of participants, with different kinds of disabilities, perceived historic heritage places as not often accessible, and many, especially those with mobility disabilities or chronic pain, spontaneously mentioned inaccessibility in their own definition of heritage. For example:

It's the history of course, but not accessible [laughs]. That's for sure. Then there's the complexity of making it accessible, given the heritage regulations. You can't do that, it has to be with the same materials. You know, you can't just do whatever you want, but I think … there's certainly a way of doing something. (P06W-Mobility)

Most participants apprehended that the sites they visited had not been built with accessibility in mind, and the complexity of adapting them. They were in favor of heritage preservation, but appreciated the sites and felt that everyone should have access to historic sites, regardless of their condition. For example, one wheelchair-user participant mentioned:

When they (historic sites) were built, they weren't adapted for people in wheelchairs. So I always think about that. But yes, a historic site, should be, uh (hesitation), should still have access to everyone. Even for people in a wheelchair. (P07M-Mobility)

Although some participants were open to partial access to heritage buildings and sites, or to alternative solutions, they preferred to have physical access to the integrity of the site: “unless we visit it virtually. But it's fun to be in person…” (P06W-Mobility).

In short, despite recognizing the importance of preserving heritage for its historical and cultural value, participants categorically expressed their interest in accessing and enjoying it.

## 4 Discussion

The purpose of this study was to explore the experiences of people with visible and invisible disabilities when visiting historic heritage sites considering accessibility issues. Most of the studies about public building accessibility do not included participants in the accessibility evaluation (39). The use of go along interviews for data collection represents a suitable method in the field of accessibility and has enabled us to obtain the perspective of the main people concerned by accessibility problems (25). Furthermore, working in this way with the people directly concerned in a real-life context encourages the creation of realistic solutions in the future (40, 41).

The content of the three themes—obstacles and impact on participation, disabling accessibility and heritage meaning—provided a portrait of the elements that have an impact on people with disabilities in a heritage context, as well as the meaning and importance of built heritage for people with disabilities. The obstacles identified by the participants are very varied in nature and intensity and differ according to the individual and the type of disability. This may be related to the heterogeneity of the study sample, which included people of different ages and genders, with various disabilities and levels of autonomy. However, there were some elements that appeared to be problematic for the majority of participants, regardless of disability type and other participant characteristics. Many of these barriers correspond with what is already known from the literature, such as uneven flooring (pavement), steps, particularly at shop entrances (9, 12, 13, 18), as well as objects on sidewalks (9, 19) and the lack of accessible toilets (13, 42). However, some participants ignored some obstacles that were obvious for the research team, such as access to certain shops or restaurants with steps at the entrance. One possible explanation of this reaction could be a coping or acceptance strategy related with the adaptation to the disability (43) in order to avoid continuous confrontation with environmental barriers It is worth noting that commercial and restaurant buildings were not originally designed as public spaces. For the most part, they were conceived as residential buildings.

The presence of many people was also central to the results of the study. Heritage places contribute to the tourist appeal of cities. When historic districts become tourist attractions, they are often crowded. These areas may not necessarily have been designed or adapted to receive a large number of visitors. This can contribute to large groups of people being an obstacle to circulation, for example. It should also be pointed out that even if the most restrictive measures of the Covid-19 pandemic has ended when the data collection began, some travel measures were still in force (e.g., vaccination proof at boarders). This could have resulted in some interviews, particularly the first ones, being conducted in a less busy context than usual. Other measures, such as mask wearing, changed or disappeared during de data collection period. Concerning the School of Architecture, located in the *Séminaire de Québec*, it also less busy than usual, particularly during the last interviews, due to students' summer vacations. It may mean that the volume of visitors and students could be perceived as less of an obstacle for some participants in the study.

Some of the obstacles mentioned by study participants have received less attention in the literature. For example, none of the articles in the reviewed literature addressed the issue of excessive stimuli in historic heritage places. These elements would seem to have a closer link with invisible disabilities (e.g., autism) than with visible disabilities (e.g., motor disabilities). However, elements such as noise or visually charged environments, which were mentioned by participants as sometimes problematic in historic heritage contexts, were also considered obstacles in other contexts (44, 45). In addition, in line with what the study participants said about heritage contexts, the presence of rest areas—quiet places where there are few sensory stimuli—would be also particularly appreciated by people with autism in other contexts, such as home or school (46, 47).

A variety of obstacles were mentioned by participants. Indeed, many elements of the physical environment were problematic, and this may be due to the characteristics and materials of the historic buildings (5). However, other obstacles were mentioned by participants, such as the presence of many people, the complexity of the buildings or sensory overload. Barrier-free design originally referred to access for wheelchair users (48). However, today, following the evolution of this field and related concepts, “universal design is a process that enables and empowers a diverse population by improving human performance, health and wellness, and social participation” (49) and accessibility aims “to ensure to persons with disabilities access, on an equal basis with others, to the physical environment, to transportation, to information and communications, including information and communications technologies and systems, and toother facilities and services open or provided to the public” (50). Both the finding of this study and current concepts of accessibility transcend barriers in the physical environment, which mainly affect people with mobility impairments and it should be taken into consideration when developing recommendations and accessibility solutions [e.g., tactile signage or auditive information could improve accessibility of historic heritage places (51)]. Participants mentioned several activities that could be limited for them in the studied areas and buildings of Old Quebec. The majority of these activities were leisure activities (e.g., going to a bar or restaurant, attending a show). According to the MDH-PPH, leisure activities are “habits related to recreational or other activities, carried out during free time in a context of pleasure and freedom” (2). According to this model, leisure includes, among other things, socio-recreational activities (e.g., going to a bar, sightseeing or visiting relatives) and also arts and culture (2). People with disabilities could therefore experience a disabling situation in certain leisure-related activities if historic heritage places are not accessible. Social relationships, sometimes cultivated during leisure activities, could also be limited.

Most participants described strategies they use to cope with the difficulties they encounter, which could enable them to counter certain obstacles. However, their strategies were not always sufficient to successfully complete activities. The results obtained with regard to limited activities are more pronounced in the go along interviews on in the Petit-Champlain and Place Royale sectors than in the *Séminaire de Québec*. Two factors could have an impact. First, the fact that the architecture school was not busy, and that some services were not available due to school vacations, could limit the possible interactions between participants and the environment. Secondly, the participants recruited were not necessarily students and might feel less identified with the environment explored at the *Séminaire de Québec*. However, the Petit-Champlain and Places Royale areas of Old Quebec were familiar to most participants and included spaces where they would like to carry out activities in a real-life context.

Regarding the accessibility solutions already present in the sites visited during the go along interviews, some seem to limit the autonomy of people with disabilities. The implementation of poorly adapted solutions may be due to a lack of awareness or resources among decision-makers (52, 53) and designers (54), as well as to current accessibility standards, which are often limited and lack a holistic approach, considering all the dimensions and needs of people with disabilities. Another factor that may influence the use of solutions that do not fully meet the needs of the people concerned could be the limitations on modifying heritage sites due to conservation laws (7). These partial solutions, while they may be useful for some people, could contribute to segregation and a feeling of exclusion among people with disabilities. As a result, people with disabilities may even avoid visiting heritage sites.

Heritage places often have historical, cultural or social significance (6) and reflect the identity of a culture (7). Lack of access to these places is likely to encourage a lack of access to cultural elements essential to the development of a socio-cultural identity among local residents. For example, according to Newman and McLean (55), lack of access to museums, often key sites for heritage and culture, could have an impact on identity development, with loss of identity concomitant with social exclusion (56). In the province of Quebec, Canada, heritage places represent a key element for the local cultural identity of the population living there. However, if an area is not accessible, this could also have an impact on tourists and their understanding of local history (57). Heritage preservation is therefore important for the development and maintenance of social identity. However, social and architectural environmental factors in heritage contexts can have an impact on the social roles of people with disabilities, such as access to culture and socio-recreational activities. This could also have an impact on this population's sense of identity and wellbeing. As Vardia et al. (58) have already documented, the balance between accessibility and conservation of the place and its ambience is fundamental. It is therefore essential to consider the needs of people with disabilities, who could also benefit from the cultural richness that heritage places can provide, and so promote the evolution of socio-cultural identity.

Given the importance of heritage sites and buildings in the lives and identities of citizens, it is necessary to think about the possibility of making compromises with respect to the physical environment, but also to the social environment. In addition to architectural solutions that can be installed in the physical environment of heritage sites, other elements should be reviewed in order to improve site accessibility. It would be relevant to review and rethink some of the norms of preservation of historic heritage places in order to make accessibility interventions that respond to a greater number of disabilities. Alternatively, the human environment could partially compensate for the lack of accessibility to some extent, as happens in other places, for example,some assistance services are usually provided in airports and train stations to improve the experience of people living with disabilities. It would be important to provide services that can provide support when possible architectural accessibility is not sufficient. For example, awareness campaigns and training, taking advantage of the willingness of people who are willing to help, and providing an official framework in which better services can be offered and not only depend on the good intentions of the individuals.

### 4.1 Strengths and limitations

The methods used in this study allows us to identify several strengths regarding its trustworthiness (29, 59). Prolonged engagement in the field and the combination of participant discourse and persistent observation during data collection, as well as team discussion and the involvement of several team members in the analyses, promote the credibility and dependability of the study (59). Preliminary findings of this study were presented to some of the participants in a co-design group as part of a subsequent stage of the study as a member checking strategy. Although the aim of the study is not to generalize the results obtained, the heterogeneous sample, including people with different characteristics regarding age, gender, and type of disability, as well as the description of the sample and context included in the article, favors the transferability of the results (59). This study also has some limitations related to the context and the methodology. First, due to difficulties in recruiting this population, only one person with an intellectual disability participated in the study, so the similarities and differences within the same population could not be explored. Then, go along interview method, particularly when used with people with disabilities, may involve some additional limitations. For example, even if the level of autonomy was not explicitly considered in the participant selection criteria and was not assessed, participants must have a relatively high level of autonomy, as the method required them to be able to navigate on a real context and communicate simultaneously. It may explain the participants' ability to develop their own strategies. Other accessibility issues might have emerged with a less autonomous sample. Although the locations were partially accessible, the itineraries were predefined, and little freedom was offered to the participants in their choices of itinerary to avoid possible safety issues and frustration that participants might experience in environments with too many obstacles. Also, predefine itineraries ensure exploration of the features of the heritage environment. Two elements could have an influence on participants' responses regarding their interest in heritage: firstly, the use of convenience sampling could have favored participation by people with a greater interest in heritage. Secondly, the data collection method used could have contributed to a desirability bias in this regard. However, desirability bias is more frequent in the study of sensitive or controversial issues and seems less likely in this study on the basis of the participants' statements. It is difficult to assess (or self-assess) interest in spaces where access is restricted to the individual, and the most salient element in this respect is the interest and right to have access to these places, independently of the interest the individual may have in history and heritage. Finally, as Ripat and colleagues (60) and Morales and colleagues (61) have shown previously, there are accessibility issues specific to winter, and others can be amplified by weather elements such as snow. However, to ensure the comfort and safety of our participants, team members decided to conduct all the go along interviews in spring and summer.

## 5 Conclusion

Access to historic heritage places remains difficult for people with disabilities, and they often encounter obstacles in the physical and social environment in this context. Sometimes, the strategies developed by people with disabilities to compensate for environmental obstacles enable them to access and carry out some activities, often in part. However, they often find themselves in situations of discomfort or risk. This can lead to inequalities in access to culture and to public spaces whose functions ensure certain fundamental rights, contributing to issues of equity for people with disabilities. The inaccessibility of heritage places and obstacles in the environment can have an impact on social participation, limiting access to culture and the fulfillment of certain activities, particularly those related to leisure and relationships with other people. Although people with disabilities often perceive historic heritage sites as inaccessible and the interest in history and culture is difficult to know because the access is limited for them, they are interested in accessing them. The development of accessibility solutions that meet the real needs of people with visible and invisible disabilities is therefore essential to fully enjoy heritage contexts, and to reduce the inequalities experienced by this population. In further research, detailed information on environmental barriers and facilitators will be reported. In addition, the results of this study will serve to co-create (41) accessibility solutions for historic heritage sites, where experiential and theoretical experts will be involved to develop realistic solutions that meet the needs of all stakeholders.

## Data Availability

The raw data supporting the conclusions of this article will be made available by the authors, without undue reservation.
